# The Y Chromosome: A Complex Locus for Genetic Analyses of Complex Human Traits

**DOI:** 10.3390/genes11111273

**Published:** 2020-10-29

**Authors:** Katherine Parker, A. Mesut Erzurumluoglu, Santiago Rodriguez

**Affiliations:** 1 Bristol Medical School, University of Bristol, Bristol BS8 1QU, UK; kp16445@bristol.ac.uk; 2 Medical Research Council (MRC) Epidemiology Unit, University of Cambridge, Cambridge CB2 0SL, UK; mesut.erzurumluoglu@mrc-epid.cam.ac.uk; 3 MRC Integrative Epidemiology Unit (IEU), Population Health Sciences, Bristol Medical School, University of Bristol, Bristol BS8 1QU, UK

**Keywords:** Y chromosome, Y haplogroups, complex locus, genetic association analyses, genetic epidemiology, complex human traits

## Abstract

The Human Y chromosome (ChrY) has been demonstrated to be a powerful tool for phylogenetics, population genetics, genetic genealogy and forensics. However, the importance of ChrY genetic variation in relation to human complex traits is less clear. In this review, we summarise existing evidence about the inherent complexities of ChrY variation and their use in association studies of human complex traits. We present and discuss the specific particularities of ChrY genetic variation, including Y chromosomal haplogroups, that need to be considered in the design and interpretation of genetic epidemiological studies involving ChrY.

## 1. Introduction

**Figure 1:**
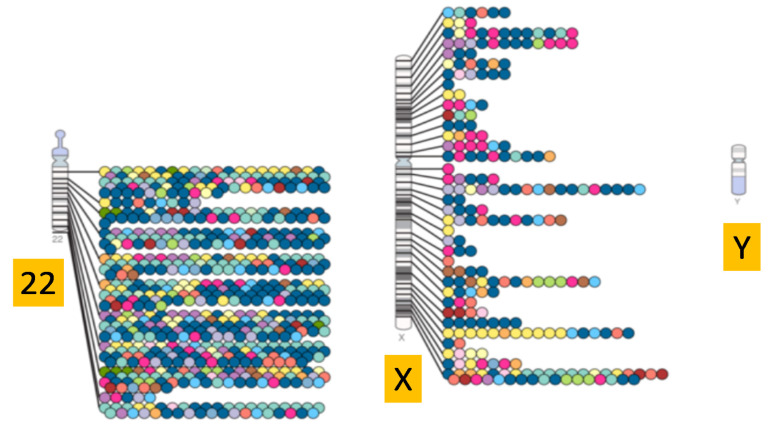
GWASs can be used to look for genetic associations with human disease traits across the human genome. This figure is a modified version of the GWAS diagram from the September 2019 version of the GWAS Catalog, a directory which contains more than 157,000 associations from 4220 publications [2]. The diagram shows hits identified on chromosomes 22 and X, with different categories of disease indicated by different coloured markers. There are no hits which correspond to ChrY. Legend has not been included for clarity, but the original diagram and legend can be found at https://www.ebi.ac.uk/gwas/diagram.

During the last decade, genome-wide association studies (GWASs) have identified large numbers of loci associated with common complex human traits, enabling a better understanding of the genetic architecture of human disease [1]. This progress is documented by the NHGRI-EBI GWAS Catalogue [2], which includes all published GWAS hits to date. The latest version (accessed 11/10/20) of the widely used diagram, “SNP-trait associations, mapped onto the human genome by chromosomal location and displayed on the human karyotype”, provided by the catalogue (Figure 1), shows evidence of many SNP–trait associations on all 22 autosomes and the X chromosome. Notably, however, none of the GWAS hits found to date reside on the Y chromosome (ChrY).

Results from studies of various designs not limited to GWASs (e.g., candidate gene association analyses) also provide evidence that minimises the role of ChrY common genetic variation in relation to human complex traits. It is important to consider alternative explanations for this lack of association, such as the inherent genetic complexity of ChrY, which differs from the remainder of human chromosomes in terms of structure, function and population history. These differences make it more complex to analyse than other human chromosomes, which consequently leads to the removal or misanalysis of ChrY variants from association studies.

This review addresses specific features of human ChrY relevant for the design and interpretation of genetic association studies of complex human traits and covers different study designs applied in genetic epidemiological studies of ChrY, discussing their strengths and weaknesses, as well as potential avenues for future research.

## 2. The Human Y Chromosome, a Complex Locus for Complex Trait Analysis

Since the discovery of ChrY in 1921 [3], specific particularities related to its structure, function and population history have been discovered. A comprehensive list can be found in Table 1.

In summary, ChrY is the sex-determining chromosome in humans and is passed strictly from fathers to sons. Current understanding suggests that the function of ChrY is limited with regards to complex disease causal genes. Here we provide a review of the literature related to ChrY and complex diseases and also make suggestions for future studies.

### 2.1. Structure of Human Chromosome Y and Recombination

In mammals, gender is dictated by either the presence or absence of ChrY. This chromosome is structurally unusual in relation to autosomes and, in humans, bears an extremely high quantity of repetitive content. ChrY also contains heterochromatic, X-transposed, X-degenerate, ampliconic and pseudoautosomal regions (PAR) [17], formed over time, as the previously autologous pair underwent a sequence of large scale inversions and deletion events [26].

**Figure 2:**
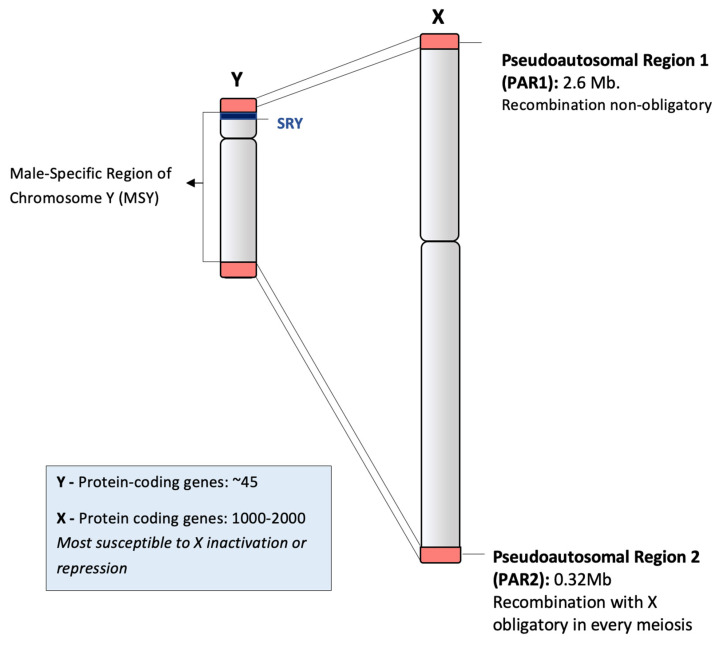
Schematic representation of the Y and X chromosomes. Pseudoautosomal regions 1 and 2, the MSY and the location of SRY are indicated.

ChrY shows similarity with its counterpart, the X chromosome, at the pseudoautosomal regions present at the telomeres (Figure 2). As a result, crossovers involving ChrY can only occur at these regions. The remainder of ChrY (~95%) is referred to as the male-specific region of ChrY (MSY) [17], previously known as the non-recombining region of Y (NRY) [11]. Variation of the MSY over generations is contributed to by *de novo* mutations and both X-to-Y and Y-to-Y gene conversion [17,27,28,29]. One study suggests an average of 600 nucleotides per ChrY in a newborn male have undergone Y-to-Y gene conversion in recent human evolution [27]. Whilst significant, this amount of variation is relatively small when compared with autosomes and, when accompanied by uniparental inheritance and lack of crossing over, means that Y chromosome lineages are relatively easier to trace back through time than autosomal lineages.

### 2.2. The Functional Role of the Human Y Chromosome

It has been frequently suggested that the functional significance of ChrY is minimal. ChrY would therefore represent a ‘functional wasteland’ and it would be destined to disappear from the nuclear genome [30]. Although absence of evidence does not imply evidence of absence for ChrY [31], this has contributed to the historical consideration of ChrY as a human chromosome with an insignificant functional role.

#### 2.2.1. Deterioration of the Y Chromosome

Current evidence suggests that deterioration of ChrY was initiated by the acquisition of a male sex-determining function early in mammalian evolution and subsequently perpetuated by repression of recombination. This lack of recombination is also partly responsible for the loss of much of the ancestral gene content, by way of a phenomenon known as Muller’s ratchet [32]. As a consequence, ChrY has acquired genes which relate to male-specific functions.

There is controversy about the consequences of ChrY deterioration. Some authors have argued that it will inevitably lead to continued degradation and eventual disappearance of the human ChrY [33]. This argument sites evidence from parallel systems of sex determination (ZW) and comparisons with other mammals [30,34,35]. In contrast, other authors site gene conversion within palindromes [17] and a relative slowing of rate of gene decay in ChrY over recent millennia as evidence supporting continuing evolution in current human populations [26,36].

Disentangling which of these opposed hypotheses better explains the recent evolution of ChrY is an active area of research [37,38]. Unanswered questions to date include (a) to what extent is direct comparison of equivalent but different genetic sex-determining systems, namely XY and ZW, appropriate? (b) Does the comparison of ChrY from long-diverged species overlook the complexities of its evolution and bring about misleading conclusions? (c) Would understanding the extent of genetic deterioration of ChrY be relevant and informative for genetic association analyses?

The degradation of ChrY may represent a biological explanation for the reduced number of functional loci on ChrY and hence a reduced number of associations found by association mapping approaches. If this degradation continues, then one would expect further loss of functional elements over time. Alternatively, it could be possible that degradation of ChrY has reached an equilibrium implying that current functional elements are important for males and will be maintained in the population over time.

#### 2.2.2. Genetic Content of the Y Chromosome

The entirety of the Chr Y was first sequenced in 2003 by Skaletsky et al. who identified 78 protein-coding genes, encoding 27 distinct proteins or protein families [17]. However, current transcript- and protein-level evidence suggests the presence of 64 protein-coding genes including genes on the PAR (Ensembl BioMart; HGNC; CCDS, all accessed 11/10/20) [39,40,41]. When the MSY is considered in isolation (i.e., the genes on PAR1 and PAR2 excluded), the region has 45 protein-coding genes (Ensembl BioMart; neXtProt v2020-07-17; both accessed 11/10/20) excluding PRYP3 and TSPY9P which are now thought to be pseudogenes (Table S1) (Human Protein Atlas, accessed 11/10/20) [42]. This number is considerably smaller than the number of genes harboured within the X chromosome (n = 852) and all other autosomes (range n = 234–2059), making ChrY the chromosome with the least number of protein-coding genes in humans (Ensembl BioMart, accessed 11/10/20).

In contrast to its lack of protein-coding genes, ChrY presents an unusually high level of structural polymorphisms. ChrY is enriched with various types of repeat polymorphisms including SINEs, endogenous retroviruses and segmental duplications [32].

#### 2.2.3. Gene Function of the Y Chromosome

The first genes identified on the MSY were found to be instrumental in initiating and maintaining the male phenotype [5,7,9] and conferring male fertility [43,44]. These findings likely contributed to the belief that the principal function of the MSY was to facilitate development and maintenance of the male phenotype, and further, that it held little consequence for health in general. Although this has been the consensus for some time, confidence in this hypothesis is dwindling. The functions of genes encoded by ChrY have been summarised by Bellott et al. [45]. These functions range from histone lysine demethylation, regulation of stem-cell self-renewal and translation, and protein modification (such as the deubiquitinase USP9Y) [45,46]. Although our understanding of MSY and the role of these genes is far from complete, it seems plausible that variation within these loci could influence fitness.

These findings have promoted a shift in thinking that has given way to an explosion of research into ChrY and numerous studies have now been published that search for associations between the MSY and male traits.

### 2.3. Specific Particularities of the MSY in Relation to Human Population History

The MSY is a powerful tool with which to investigate human population history [47,48,49,50,51,52]. In order to design appropriate genetic association studies of human complex traits, one must possess an appreciation of the specific particularities of the MSY. This fundamental requirement may also lead to the discovery of novel applications of the MSY, promoting a deepening of our knowledge of human population history and structure in relation to genetic architecture of human traits.

Firstly, the haploid nature of ChrY means that it has a smaller effective population size when compared with either autosomes or the X chromosome, which have four times and three times greater effective population sizes, respectively. As a result, ChrY is much more vulnerable to the effects of drift (already a key dictator of the evolution of ChrY due to lack of recombination), which results in the accentuation of the differentiation between populations from one generation to the next [53,54].

Secondly, the perspective offered by studying ChrY is representative of only males within a population. This is comparable to reasoning applied when using mitochondrial DNA (mtDNA), which is (thought to be) predominantly inherited through the female line [55,56,57]. A plethora of demographic and sociocultural practices between males and females (for example patrilocality versus matrilocality, polygamy and disparity in life expectancy [58]) have the potential to influence gene flow in the population. If ChrY is considered in isolation, intricacies of population dynamics may be missed [59]. Combining two data types (that of MSY and mtDNA) is considered more efficacious. This contextual approach to the analysis of the MSY and its variation is in itself another application for the MSY, which has allowed collation of more information about the variation in movement between males and females over time [60,61,62].

Thirdly and importantly, there are key assumptions upon which many of the investigations mentioned above are based—including that these markers are neutral. This assumption is being tested in recent research which looks at whether variation in the MSY could confer significant differences in fitness or affect complex traits [15,63]. In addition, Y chromosome data can be used to estimate the time to most recent common ancestor (TMRCA) and, due to its inherently smaller population size, the estimation given by using the Y chromosome should be proportionally more recent than the time indicated by neutral markers found on autosomes or the X chromosome [54]. TMRCA estimates based on ChrY are revised as the knowledge in the field increases. For example, it has been suggested that TMRCA estimates given by ChrY data are even more recent than would be predicted and thus provide possible evidence of natural selection acting on ChrY [54,64]. In fact, there is recent evidence suggesting that natural selection is involved in the maintenance of genetic variation involving copy number variants [65]. This is an area where some controversy remains, following the discovery of a new lineage (haplogroup A00) in 2013, revisions of the estimated TMRCA have been suggested [66,67,68,69].

## 3. Y Haplogroups

**Figure 3:**
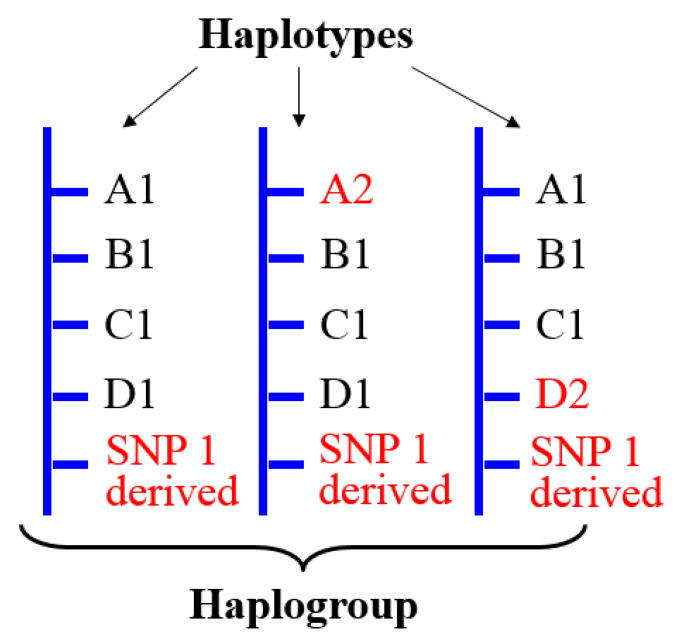
Haplogroups are groups of haplotypes that share a common ancestor. Individuals belonging to a specific haplogroup share the same derived allele for one informative SNP. A derived allele is a new variant in a locus, different from the original non-mutated allele (which is known as the ancestral allele). This figure shows three haplotypes. All of them share the derived allele for SNP1. Variation in other SNPs (in red) creates different haplotypes within this haplogroup.

A key particularity of the MSY in relation to human population history is the existence of Y haplogroups. This source of genetic variation, absent on autosomes, is created by the absence of recombination on the MSY. Y haplogroups are defined as groups of similar Y haplotypes (genetic markers that are inherited together) that share a common ancestor with a particular SNP mutation (Figure 3).

Y haplogroups have been identified and organised into a phylogenetic tree which is added to and amended as new ChrY SNPs are discovered [16,70]. Currently, more than 300 haplogroups have been identified throughout the world and have been studied as a means to investigate and understand several aspects related to Genetics. Their use in genetic genealogy and forensics has been discussed at length in recent reviews [71,72]. We briefly discuss here the use of Y haplogroups in phylogenetics and population genetics.

The use of Y haplogroups as a tool for informing investigation into early human and indeed primate population history has been widely studied. By looking at common Y haplogroups and the variation which they contain, it is possible to estimate the time of origin and hypothesise about the movement of their carriers across the globe. To this end, climatic and archaeological data have been analysed in conjunction with MSY insertion and deletion events [51,73,74], microsatellites [75] and minisatellites [76]. Analysis of the MSY has provided solid evidence about the origins and early migration of *Homo sapiens* [47,77,78]; the identification of candidate founder haplotypes for numerous populations [79] and the elucidation of intricacies of inter and intrapopulation dynamics across the globe, including suggestions of partition and coalescence events and previously unknown genetic bottlenecks [74,80].

In addition to the investigation of human population history, the MSY has been used to gather information about more recent human demographics. Analysis has allowed the identification of Y haplotype clusters and their distribution throughout Europe [81,82,83,84,85]. These subdivisions within the population are a potential source of variation in disease risk and further studies incorporating this aspect of variation could have important implications for using haplogroup data in the study of disease.

## 4. The Y Chromosome in Genetic Epidemiological Studies of Human Complex Traits

Genetic epidemiological studies involving ChrY aim to analyse the role of ChrY and environmental factors in determining or influencing health and disease in families and in populations. To date, a variety of human complex traits including behaviour and psychiatric traits [24], cancer risk [86,87], autoimmunity [88,89] and HIV progression [23] have been interrogated to this end, but the relationship between the human ChrY and cardiovascular risk is one of the best studied examples. There is a recent review [90] covering many aspects of this relationship, and therefore, we will not cover details here. Instead, we will present key aspects for consideration when utilising ChrY in genetic epidemiological studies using studies that explore cardiovascular risk as examples. We will discuss gender differences in relation to disease incidence and progression, study design and the use of Y haplogroups in association mapping.

### 4.1. Gender Differences in Relation to Disease

The fact that females lack all ChrY-specific genes implies that ChrY does not contain genes essential for survival. However, gender differences in disease incidence have been documented for many conditions [91,92,93]. For example, females are more likely to suffer from certain systemic autoimmune conditions such as systemic lupus erythematosus [94] and rheumatoid arthritis [95]. Conversely, men are more likely to suffer from schizophrenia [92] and cardiovascular disease [93]. There are also significant gender differences in progression of certain diseases [96,97]. It is likely that an interplay of genetic and environmental factors explains these differences. Variables that have been identified thus far include gender differences in lifestyle patterns (e.g., alcohol consumption, smoking and dietary habits) [98], variation in hormonal levels during development [99], hormonal influence during adult life [100] and many others such as pregnancy [101].

### 4.2. Study Design

Study design is key for the success of association mapping analyses [102]. The association mapping study designs utilised to date to explore the relationship between genetic variation on ChrY and human complex traits include linkage studies, candidate gene studies, animal models and genome-wide association studies (GWASs). Table 2 summarises different aspects of each of these study designs and examples where they have been applied to disentangle the genetic contribution of the MSY in relation to human complex traits. Table 2 also shows the implicit assumptions of each study design, their strengths and limitations and examples of published studies to help contextualise the contribution of these association mapping approaches to current understanding of the relationship between MSY and complex traits.

#### 4.2.1. Genome-Wide Association Studies (GWASs)

Table 3 shows a summary of issues relevant to genetic association studies carried out using MSY SNPs (including GWASs). These include statistical power, linkage disequilibrium patterns, population stratification, presence of complex loci, colocalisation, pleiotropy, gene–gene interactions, gene/protein expression and replication.

Although these aspects are also relevant to genetic association studies involving SNPs located on autosomes, studies involving ChrY need to account for additional issues that are specific to the genetic complexities of the MSY. These specific characteristics of ChrY open additional effects and possible solutions to account for them in the design and interpretation of GWASs involving ChrY SNPs. Table 3 summarises the implications of each of them on the interpretation of genetic association studies. We also point to possible solutions to minimise their impact.

The current GWAS literature does not offer conclusive evidence for a role for the MSY in disease susceptibility. Various potential reasons for this exist. For example, ChrY is excluded from most GWASs. In fact, ChrY SNPs are excluded from some commonly used genotyping arrays [112]. Another reason is that some authors opt for to exclude ChrY SNPs from GWAS analyses. Of note however, is that ChrY is routinely excluded from GWASs as all common variants in the MSY are effectively in linkage disequilibrium (Table 3). Although more recently X has been more likely to be included [113], this practice has left a dearth of evidence with respect to ChrY. For example, out of more than 50 GWASs conducted over the last 10 years on chronic kidney disease, only one reported results of ChrY analyses [112].

#### 4.2.2. Expression Quantitative Trait Loci (eQTLs) and Protein Quantitative Trait Loci (pQTLs)

Currently, there are 45 protein-coding genes specific to the Y chromosome (i.e., excl. genes in the pseudoautosomal regions; Ensembl BioMart, accessed: 11/10/20). These 45 genes code for 27 distinct proteins/protein complexes [114].

If ChrY variants are included in a GWAS, any identified associations will need to be biologically plausible. To aid the search for prime candidates for GWASs of common complex diseases, we queried the Human Protein Atlas (v19.2; Ensembl v92) for all 45 genes [121] (using aliases from Ensembl BioMart database [122] to make sure we captured all) in all tissues except the testis and female-specific tissues (i.e., vagina, ovary and fallopian tubes). We also removed results marked with "not detected"—indicating that the levels of expression, if any, were below the limit of detection. As a positive control, we queried to confirm whether the proteins were detected in the testis; 27 distinct proteins were detected (validated by transcript-level evidence): DDX3Y, ZFY, RPS4Y1, VCY1B, VCY, CDY2B, TSPY2, RBMY1F, CDY1, CDY1B, CDY2A, UTY, DAZ3, DAZ1, EIF1AY, DAZ4, DAZ2, RBMY1J, TSPY3, TSPY8, TSPY4, RBMY1A1, TSPY10, RBMY1E, RBMY1B, RBMY1D, and TSPY1.

**Figure 4:**
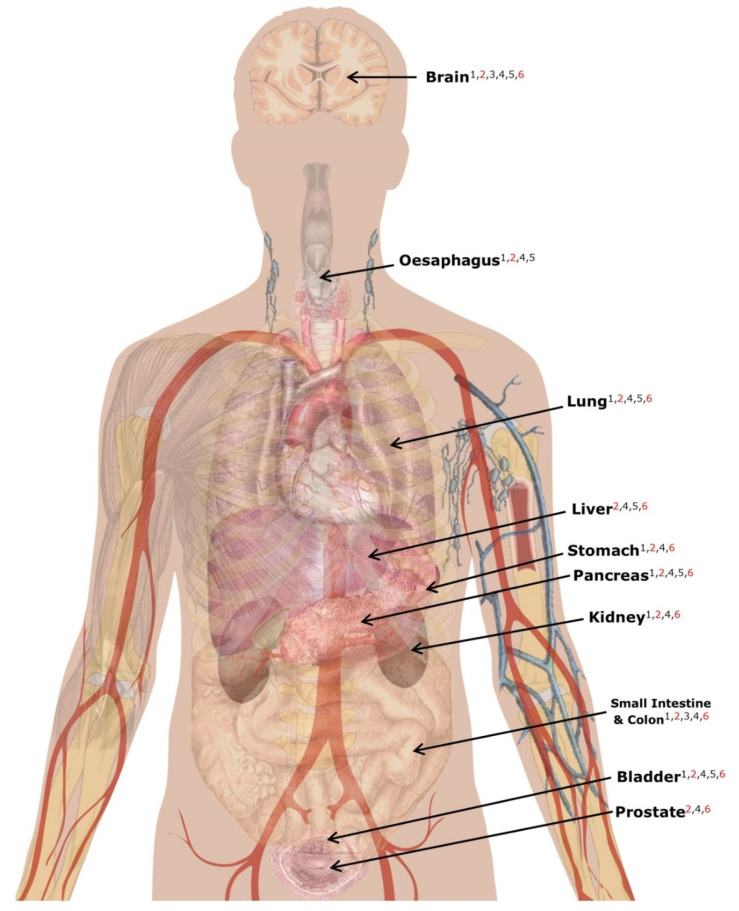
Protein-coding Y-chromosomal genes expressed in non-gonadal tissues (not comprehensive) as detected by the Human Protein Atlas. Protein labelled 1: DDX3Y; 2: EIF1AY; 3: NLGN4Y; 4: RPS4Y1; 5: UTY; 6: ZFY. Proteins highlighted in red have a low quality of supporting evidence. Image source: commons.wikimedia.org/wiki/Human_body_diagrams (Wikimedia CC0 licence).

The only proteins that were detectable outside of the testes by the Human Protein Atlas were DDX3Y, ZFY, RPS4Y1, NLGN4Y, UTY, and EIF1AY, which would make them and eQTLs/pQTLs affecting their expression prime candidates for non-fertility related diseases/traits (Figure 4). There are three more proteins—KDM5D, USP9Y, TMSB4Y—which are putatively ubiquitously expressed as determined by RNA-seq carried out by GTEx [114] but are not yet validated by the Human Protein Atlas’ immunohistochemistry assays – and would thus also be candidates for non-fertility related diseases/traits [63,114].

We further searched the literature (e.g., GTEx [114], BRAINEAC [123], and Blood eQTL server [124]) to identify eQTLs on the Y chromosome. However, we could not find any—reflecting the need for further research in this area (Table 3).

When interpreting these findings, one must consider the limitations of the analysis carried out by the Human Protein Atlas, which uses an antibody-detection method, inevitably dependent on the accuracy of antibodies used [125]. In some cases, antibodies are not able to distinguish between proteins in the same family [125]. For example, antibodies may fail to distinguish between RBMY1A1 and RBMY1B, two members of the same family, both located on ChrY; similarly, between E1F1AY and E1F1AX which are located on ChrY and ChrX, respectively. However, the Human Protein Atlas uses other lines of evidence for antibody verification, including transcript mRNA validation (https://www.proteinatlas.org/about/antibody+validation). Secondly, investigation here is limited to those genes that are ubiquitously expressed, there are other protein-coding genes whose proteins have been detected outside the gonads but not ubiquitously which may be plausible targets for complex trait analysis. For example, PCDH11Y, predominantly expressed in the brain [63], which could perhaps influence brain related diseases or behavioural traits. Thirdly, as protein expression varies with development, there may be ChrY gene expression which affects complex trait development, which is undetectable by currently used methods because they are not expressed in adult tissues [114].

### 4.3. Use of Y Haplogroups in Genetic Association Studies of Common Complex Traits

Y chromosome (Y-DNA) haplogroups are more widely used in population genetics than in genetic epidemiology, although associations between Y-DNA haplogroups and several traits, including cardio-metabolic traits and psychiatric traits have been reported [20,24,110].

In contrast, non-recombining genetic variation, such as Y chromosomal (Y-DNA) haplogroups, has rarely been considered in the design and interpretation of genetic association studies [82]. We have recently studied weather hidden stratification and/or differential phenotypic effects by Y-DNA haplogroups could exist [118]. Y haplogroups can be used to stratify individuals in genetic association analyses. To this end, we followed a two-stage approach. Firstly, we stratified individuals according to their Y haplogroups and then tested for association between 32 autosomal BMI-related SNPs from Speliotes et al. [126] and BMI within each haplogroup after adjusting for the top 10 genetic principal components. Although we found evidence suggestive of an interaction between haplogroup I and *FTO* SNPs in ALSPAC, we could not fully replicate these findings in the 1958 Birth Cohort. Further studies are needed.

Consideration of haplogroups in the design and interpretation of genetic association studies of ChrY, could be a crucial and commonly omitted source of complexity. Potential caveats and limitations are in addition to those listed on Table 3 and relate to novel issues on genetic complexity, population structure and statistical power.

#### 4.3.1. Genetic Complexity Inherent to Y Haplogroups

Y haplogroups are complex genetic variants that combine haplotypes sharing a common ancestor [70]. As a consequence, individuals sharing the same haplogroup are genetically heterogeneous in relation to single genetic common variation, including SNPs. This may have direct implications for genetic association studies. A SNP signal identified in GWASs could be diluted or more difficult to identify when studying Y haplogroups. Similarly, comparing individuals from different cohorts that share the same haplogroup could represent an additional layer of heterogeneity. It is likely that such individuals differ genotypically in relation to a number of SNPs, despite belonging to the same haplogroup. This heterogeneity needs to be taken into account in the interpretation of results observed from different populations, traits and haplogroups.

Alternatively, haplogroups could be well placed to capture the effect of combinations of two or more SNPs. In fact, haplotypes can define functional units of genes [127]. Advantages of analysing haplogroups in association studies would include the fact that genetic variation in populations is organised into haplotypes and that combining SNPs into haplogroups reduces the dimension of association tests and may augment statistical power.

#### 4.3.2. Statistical Power Issues in Studies Involving Y Haplogroups

The increase in power resulting from combining SNPs into haplogroups has a differential effect on different haplogroups. An illustration table from [24] shows the frequency of different haplogroups observed from a European cohort. 72.1% of individuals belong to haplogroup R, the commonest haplogroup in Europe, 19% to haplogroup I. The remaining 9% of individuals belong to 10 other haplogroups. As a result, there is considerable within-study power heterogeneity for different haplogroups. This is a common feature of most studies involving Y haplogroups.

This has implications for study design and interpretation. Some analyses combine these heterogeneous haplogroups into a new group, whereas other simply remove them from the analyses. Collapsing different haplogroups is not an optimal solution, since different mutations are likely to be associated with a specific haplotypic background.

On occasion, studies report suggestive associations involving low-frequency haplogroups. The inherent low power of these associations together with the aforementioned heterogeneity among populations make replication of these signals difficult. This also applies to subgroups of common haplogroups. Refining associations within a haplogroup by considering different subgroups (e.g., R1a1, R1b1, R1b1b2, R1b1b2g, R1b1b2h…) mirrors the power issue that we have previously shown.

#### 4.3.3. Population Structure Particularities Specific to Y Haplogroups

Population samples of Y haplogroups present specific idiosyncrasies, different from population samples of SNPs, that need to be taken into consideration in the design and interpretation of genetic association studies.

Extensive population evidence shows that Y haplogroups (and specifically, genetic trees that generated them) describe ChrY correctly from a population point of view. Evolutionary history of Y haplogroups correlates with geography. The question, still unresolved, is whether this also creates an association to biology, specifically, to genetic association with human complex traits. Interestingly, a recent study which investigated the pattern of methylation amongst some Y haplogroups [128] has identified haplogroup-specific methylation sites accompanied by SNPs, irrespective of geographical origin. These results also prompt one to question whether evolutionary history of ChrY could correlate with human complex trait variation independent of geography.

## 5. Conclusions

To date, there is little evidence supporting a role of human ChrY variation on human complex traits. A possible reason for this is that ChrY variation is actually irrelevant in relation to human complex traits. Alternatively, one could argue that the lack of evidence could be related to study design and interpretation issues. Here, we have reviewed and presented here evidence that highlights the complexities of this issue and disentangles relevant variables and their possible role.

## Figures and Tables

**Table 1:** Chronological order of the discovery of the particularities of the Y chromosome from the discovery of the male-determining region to exploration of gene function.

Year	Description	References
1985	ChrY utilised for evolutionary studies ‘genetic distance’ genealogy	[4]
1986	First evidence that the male-determining region is located on the short arm of the Y chromosome	[5]
1989	ChrY polymorphisms utilised in phylogenetics	[6]
1990	First gene, sex-determining region Y (SRY), mapped onto ChrY	[7]
1990	Animal models used to investigate the influence of ChrY on hypertension	[8]
1991	Sry used in animal models to form transgenic mice	[9]
1997	ChrY utilised for forensic science and paternity testing	[10]
2000	ChrY utilised for phylogenetics	[11]
2000 onwards	Association between hind III restriction fragment polymorphism and cardiovascular disease detected	[12,13,14,15]
2002	Y-chromosomal haplogroups established	[16]
2003	MSY first sequenced	[17]
2005 onwards	Unconvincing evidence relating the MSY to cardiovascular disease risk is reported	[18,19,20,21]
2009 onwards	Y haplotypes utilised to investigate association between ChrY and other complex traits	[22,23,24]
2016	Exploration of the function of genes identified on within MSY	[25]

**Table 2:** Basis of different study designs used to investigate the association between ChrY and complex traits, with strengths and limitations for consideration when analysing findings.

Study Design	Basis	Strengths	Limitations	Reference
Linkage analysis	First-degree relatives are compared in order to ascertain the potential for a genetic component of disease susceptibility	1- Successful in the identification of highly penetrant genetic variants related to Mendelian traits or monogenic disorders	1- Limited application for complex traits due to the use of individuals that share similar genetic and environmental constituents, establishing true effects in multifactorial traits is limited	[44]
Candidate gene studies	A particular gene is studied based on biological plausibility. Variation at this gene is investigated in genetic association studies	1- Highly specific for genetic variation which focuses on the MSY	1- Locus selected in absence of understanding of its function and potential effects 2- Highly vulnerable to chance making conflicting evidence more likely	[12,13,14,19,20,21,103]
Animal models	Animal studies are used as a framework for looking at human disease	1- Allows careful control and manipulation of both genetic and external environment to isolate the effects of the MSY	1- Application of animal models to human disease makes two key assumptions that may be incorrect: - -That disease process studied is the same in humans as in other animals - -That the MSY in other animals and that in humans is equivalent 2- Application of studies using consomic strains is limited due to confounding factors	[104,105,106,107]
GWAS	Case-control study design used to look for common genetic variants more frequently identified in those with particular diseases	1- Allows genotype-first analysis of the MSY for which understanding of genetic content is limited	1- Sex chromosomes are routinely excluded from these study types as the entirety of the MSY is effectively in linkage disequilibrium 2- Haplotypes and haplogroups used as a basis for this type of analysis may be flawed 3- Frequency of haplotypes within haplogroups in different cohorts may be sufficiently different to dilute or exaggerate relationships seen in other populations	[15,18,22,23,108,109,110,111]

**Table 3:** Considerations and possible solutions for genetic association studies carried out using SNPs in the MSY.

GWAS Features	Y Chromosome vs. Autosomes	Implication	Possible Solution(s)	References
**Statistical power**	Only men inherit a Y chromosome	~50% reduction in sample size	1- Include the Y chromosome in GWASs—could be mandated by funders 2- Share GWAS summary results (e.g., via LD Hub, GWAS Catalogue) 3- Increase sample sizes	[2,112,115]
**LD structure**	All the common variants in the MSY are in LD	Identifying the causal variant is very difficult	1- Larger sample sizes 2- Fine mapping by (i) sequencing the MSY and the associated region, (ii) carrying out transethnic studies and/or (iii) Y-DNA haplogroup association studies 3- Functional analyses (e.g., single SNP editing)	[15,24,116]
**Population stratification**	Principal components calculated using autosomal SNPs are not applicable	Potential overadjustment and loss of statistical power	1- Sensitivity analyses with and without genetic principal components 2- Sensitivity analyses with and without Y-DNA haplogroup information as a covariate in (male-only) GWASs and looking for SNPs with significant differences in effect sizes	[117,118,119]
**Complex loci**	Many highly variable regions and repetitive sequences	Variant calling may not be accurate	Only include variants called with high confidence	[17]
**Colocalisation of eQTL and GWAS signals**	No eQTLs identified for the Y chromosome	GWAS-eQTL colocalisation analysis cannot be carried out at present	Initiate trans-ethnic eeGWASs and/or study rare variants on the Y chromosome	[114]
**Pleiotropy**	No conclusive GWAS signal identified	Not much information to link potential findings with other biological pathways	1- Initiate a consortium to identify associations on the MSY 2- Carry out PheWASs using all Y chromosomal SNPs (incl. rare variants)	[2]
**Gene–gene interactions**	Very few examples identified in autosomes. None with SNPs on the Y chromosome	Almost no statistical power to detect small effects	Hypothesis driven approaches (e.g., between SNPs associated with obesity/CVD and SNPs in/near *UTY*—a gene expressed in non-gonadal tissues)	[120]
**Gene/protein Expression**	Many MSY genes/proteins are not expressed at detectable levels in non-gonadal tissues	Identified associations are likely to be biologically implausible if not expressed in disease-relevant tissue	Query the Human Protein Atlas to check whether the putatively causal gene/protein is active in a relevant tissue (Figure 4)	[121]

## Supplementary Materials

The following are available online at https://www.mdpi.com/2073-4425/11/11/1273/s1, Table S1: Characteristics of the 45 protein-coding genes located on the human Y chromosome.
